# Impacts of eutrophication on microbial community structure in sediment, seawater, and phyllosphere of seagrass ecosystems

**DOI:** 10.3389/fmicb.2024.1449545

**Published:** 2024-08-14

**Authors:** Wenchao Deng, Shunyang Chen, Shiquan Chen, Bingpeng Xing, Zhuhua Chan, Yao Zhang, Bin Chen, Guangcheng Chen

**Affiliations:** ^1^Third Institute of Oceanography, Ministry of Natural Resources, Xiamen, China; ^2^Observation and Research Station of Coastal Wetland Ecosystem in Beibu Gulf, Ministry of Natural Resources, Beihai, China; ^3^Key Laboratory of Marine Ecological Conservation and Restoration, Ministry of Natural Resources, Xiamen, China; ^4^Hainan Academy of Ocean and Fisheries Sciences, Haikou, China; ^5^State Key Laboratory of Marine Environmental Science, College of Ocean and Earth Sciences, Xiamen University, Xiamen, China

**Keywords:** seagrass ecosystem, microbial diversity, phyllosphere microbial community, co-occurrence network, eutrophication

## Abstract

**Introduction:**

Seagrass-associated microbial communities play a crucial role in the growth and health of seagrasses. However, like seagrass meadows, seagrass-associated microbial communities are often affected by eutrophication. It remains unclear how eutrophication influences the composition and function of microbial communities associated with different parts of seagrass.

**Methods:**

We employed prokaryotic 16S rRNA gene high-throughput sequencing combining microbial community structure analysis and co-occurrence network analysis to investigate variances in microbial community compositions, potential functions and complexities across sediment, seagrass leaves, and seawater within different eutrophic areas of two adjacent seagrass meadows on Hainan Island, China.

**Results:**

Our results indicated that microbial diversity on seagrass leaves was significantly lower than in sediment but significantly higher than in seawater. Both sediment and phyllosphere microbial diversity showed no significant difference between the highly eutrophic and less eutrophic sites in each lagoon. However, sediment microbial diversity was higher in the more eutrophic lagoon, while phyllosphere microbial diversity was higher in the less eutrophic lagoon. Heavy eutrophication increased the relative abundance of phyllosphere microorganisms potentially involved in anaerobic metabolic processes, while reducing those responsible for beneficial functions like denitrification. The main factor affecting microbial diversity was organic carbon in seawater and sediment, with high organic carbon levels leading to decreased microbial diversity. The co-occurrence network analysis revealed that heavy eutrophication notably reduced the complexity and internal connections of the phyllosphere microbial community in comparison to the sediment and seawater microbial communities. Furthermore, ternary analysis demonstrated that heavy eutrophication diminished the external connections of the phyllosphere microbial community with the sediment and seawater microbial communities.

**Conclusion:**

The pronounced decrease in biodiversity and complexity of the phyllosphere microbial community under eutrophic conditions can lead to greater microbial functional loss, exacerbating seagrass decline. This study emphasizes the significance of phyllosphere microbial communities compared to sediment microbial communities in the conservation and restoration of seagrass meadows under eutrophic conditions.

## 1 Introduction

Seagrasses are considered one of the most important coastal habitats due to their support for a wide range of keystone marine species from different trophic levels. However, they are facing decline as a result of natural causes and human-induced activities (Bujang et al., 2018; Ramesh et al., 2019). Human activities, such as untreated sewage discharge and fertilizer runoff, which are rich in nutrients, contribute to the loss of seagrass (Bujang et al., 2006; Ugarelli et al., 2017), emphasizing the need to understand mechanisms affecting seagrass health under human influence to prevent further decline. Microbial communities are essential for maintaining a healthy ecosystem by playing important roles in several ecological processes, such as the oxidation of organic carbon, denitrification, nitrification, and sulfur oxidation and reduction (Nealson, 1997; Cardinale et al., 2012). Seagrass-associated microbial communities, found around the rhizosphere and phyllosphere, interact closely with seagrass, contributing to its growth (Ugarelli et al., 2017). For instance, rhizosphere microorganisms can provide inorganic nutrients to seagrass by degrading seagrass-derived organic matters (Evrard et al., 2005) and fixing nitrogen gas, especially in oligotrophic seagrass beds where nitrogen is limited (Patriquin and Knowles, 1972).

The phyllosphere refers to the above-ground surface of a plant leaves, serving as habitat for microorganisms (Vogel et al., 2021). Recent studies have revealed that the phyllosphere of seagrasses hosts rich and diverse microbial communities (Ugarelli et al., 2018; Vogel et al., 2021). These communities are often dominated by various groups capable of diverse metabolic functions, ranging from aerobic respiration to nitrogen fixation and fermentation (Agawin et al., 2016; Crump et al., 2018; Chen et al., 2022), thus supporting or inhibiting the health and growth of their hosts (Ugarelli et al., 2017; Crump et al., 2018). Though both sediment/rhizosphere and phylloshpere microbial communities are essential for the health and growth of seagrass, sediment/rhizosphere microbial communities have received enormous and widespread attention. Martin et al. (2020) have suggested that rhizosphere microorganisms could be treated as indicators of seagrass health. However, the microbial communities on seagrass leaves have been much less studied.

The physicochemical environments within a seagrass meadow ecosystem vary significantly among sediment, seagrass leaves, and seawater. Consequently, environmental changes are likely to have distinct effects on microbial communities in these different habitats. Previous investigations of seagrass-associated microbial communities have solely focused on the communities themselves (Liu et al., 2018; Zhang et al., 2020; Vogel et al., 2021), while minimal information is available about the differences in the impact of environmental changes on microbial communities across different habitats within seagrass ecosystems. Recent studies that simultaneously examined microbial communities associated with different habitats within a seagrass ecosystem found distinct differences among them due to environmental variations (Ugarelli et al., 2018; Rabbani et al., 2021; Banister et al., 2022). For instance, phyllosphere microbial communities were influenced by environmental factors such as temperature, water depth, salinity, and light (Vogel et al., 2020, 2021). In comparison, root and rhizosphere microbial communities in the seagrass meadow near a volcanic CO_2_ vent were more significantly affected by pH (Banister et al., 2022).

Human activities inducing eutrophication can affect the health of seagrass through various mechanisms such as light reduction, ammonium toxicity, water-column nitrate inhibition, and algal blooms (Burkholder et al., 2007; Dolbeth et al., 2011). Consequently, the health condition of seagrasses affects the community structure and function of microorganisms within seagrass meadows (Liu et al., 2018; Martin et al., 2020; Wang et al., 2020). For instance, the relative abundances of potential pathogen groups in seagrass meadow sediment near nutrient sources were significantly higher than in sediment further from these sources (Liu et al., 2018). However, the effects of eutrophication on the seagrass phyllosphere microbial community are largely unknown, leading to a knowledge gap about the distinct effects of eutrophication on sediment, phylloshpere, and seawater microbial community structures.

**Figure 1:**
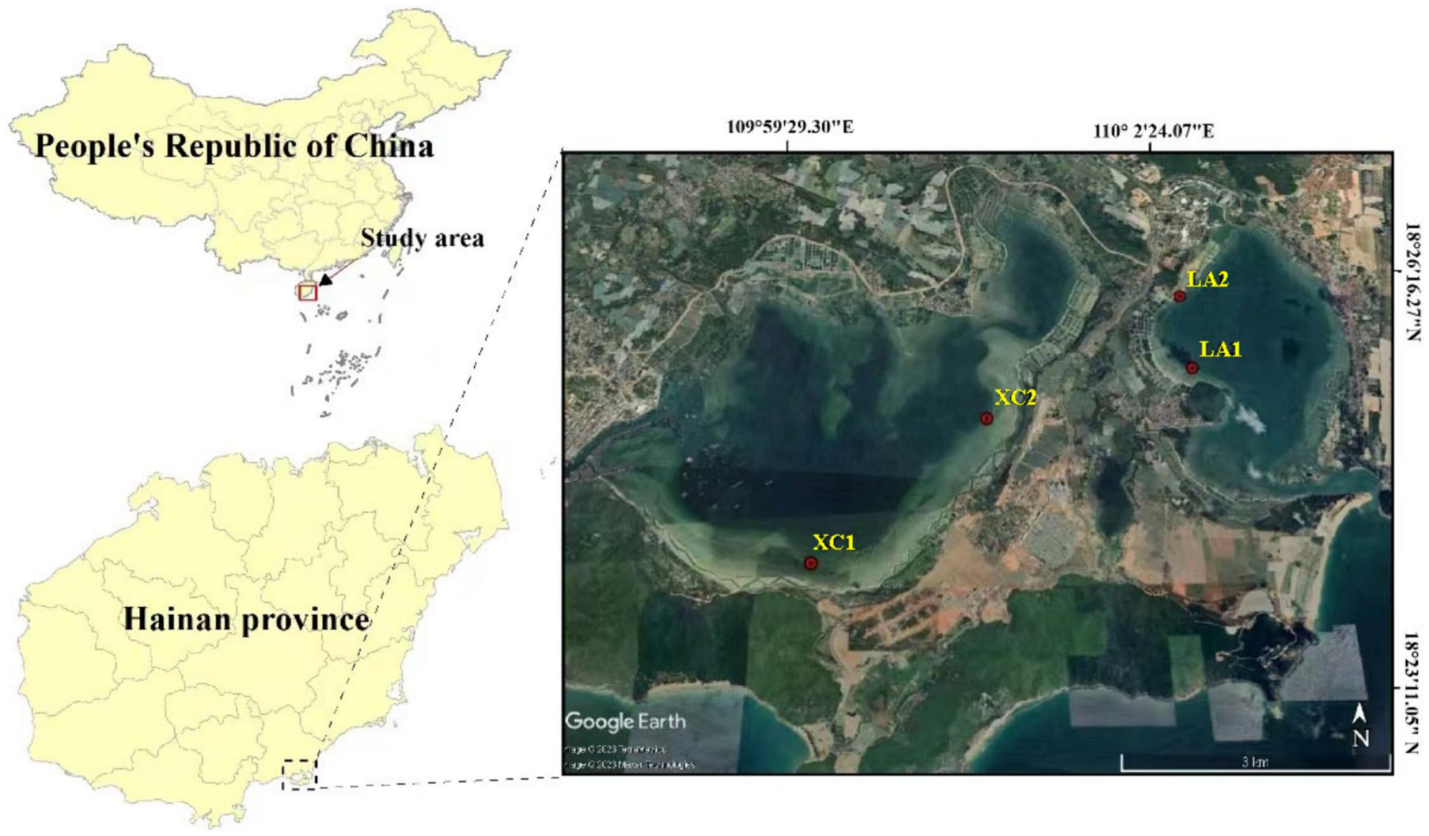
Map of sampling sites in Xincun and Lian lagoons in Hainan Island, South China Sea.

The Xincun (XC) and Lian (LA) bays are adjacent lagoons located in the southeastern part of Hainan Island, China (18°23′ to 18°26′, 109°58′ to 110°03′) (Figure 1). Both are natural, nearly closed lagoons with extensive, contiguous seagrass meadows distributed inside, serving as important fishing ports and mariculture areas in Hainan province. However, factors such as enclosed aquaculture, increased fishing activities and land-based wastewater pollution have degraded the ecological environment of both lagoons. Moreover, large amounts of bait are released from fish farms, which release organic matter and consume oxygen in the water, resulting in severe eutrophication of the water bodies. Additionally, weak water exchange between the lagoons exacerbates the eutrophication problem. This eutrophication poses a severe threat to the growth of seagrass populations in the lagoons (Li et al., 2022). To prevent further environmental degradation, fish farming has been banned in the LA lagoon, whereas it remains active in the XC lagoon. Extensive seagrass mortality has been observed along the southeast coast of the XC lagoon, with most of the seagrass leaves fallen off and in a state of decay.

In this study, we investigated the microbial community structure in sediment, seagrass leaves, and seawater habitats in the XC and LA lagoons, with the aim of exploring variations in the impact of eutrophication on phyllosphere microbial diversity, composition, and potential functions compared to sediment and seawater microbial communities within seagrass ecosystems. We also aimed to assess the complexity of the microbial community in each habitat through co-occurrence network analysis, and to examine the interactions of microbial communities from the three habitats under various eutrophication levels. We hypothesized that eutrophication may have greater effects on the diversity and complexity of phyllosphere microbial community than sediment microbial community, as phyllosphere microbial communities are more exposed to eutrophic substances in seawater compared to sediment microbial communities. Those, in turn, weaken the ability of the phyllosphere microbial community to help seagrass cope with environmental changes. This work will helps us understand the microbial mechanisms by which eutrophication affects seagrass health and enhances our recognition of the importance of phyllosphere microbial communities.

## 2 Materials and methods

### 2.1 Study area description and sampling

In the XC lagoon, seagrass is mainly distributed in the southern and eastern parts, forming continuous patches, while sporadic distribution is observed in other areas. In the LA lagoon, seagrass is primarily found in the western and southern regions, where it is extensively distributed. In the present study, two sampling sites in the lagoons of XC and LA were chosen, respectively (Figure 1). The sites XC1 and XC2 located in southern and northeastern areas of the XC lagoon, and LA1 and LA2 are in the northwestern area of the LA lagoon. The site2 (XC2 or LA2) was observed more polluted than their counterpart site1 (XC1 or LA1) in each lagoon, respectively. At each site, 100 g of sediment was collected from the surface of seagrass meadow in June 2022. Additionally, leaves of *Enhalus acoroides*, one of the dominant seagrass species in the two bays (Zhang et al., 2020), were collected near the sediment sampling location. Furthermore, 500 mL of seawater above 0.2 m of the seagrass leaves was collected. The microbes in seawater were filtered on a 0.22 μm-pore size polycarbonate filter (Millipore Corp., Merck KGaA, Germany). All samples were taken in triplicate. The sediment, seagrass leaves, and the polycarbonate filters were immediately flash-frozen in liquid nitrogen upon collection and subsequently stored at −80°C.

### 2.2 Physicochemical analysis

The seawater temperature, salinity, and pH were determined *in situ* using a thermometer, a portable refractometer, and a pH meter (WP-81, TPS, Banksia Scientific Co., Brisbane, Australia) respectively. Seawater NH_4_^+^, NO_3_^−^, and NO_2_^−^ concentrations were measured according to methods described by Deng et al. (2021) with a four-channel continuous Flow Technicon AA3 Auto-Analyzer (Bran-Lube GmbH, Norderstedt, Germany); the concentration of labile phosphate (LP) was determined using the phosphomolybdenum blue spectrophotometric method with a UV–visible spectrophotometer Evolution^™^ 350 (Thermo Scientific, Waltham, United States) (He and Honeycutt, 2005); chemical oxygen demand (COD) and dissolved oxygen (DO) were measured using alkaline potassium permanganate method (Rho et al., 2018) and iodometric titration method, respectively, with a titrator; chlorophyll *a* (Chl*a*) was determined using spectrophotometry method (Aminot and Rey, 2000). For sediment chemical parameters, organic carbon (OC) and total nitrogen (TN) contents were analyzed using a TOC analyser coupled with a nitrogen analyser (Vario TOC Cube, Elementar Analysensysteme, Langenselbold, Germany) after acidification to remove carbonates (Zheng et al., 2023); total phosphorus (TP) content was measured using spectrophotometer method with a UV–visible spectrophotometer Evolution^™^ 350 (Thermo Scientific, Waltham, United States) (Aspila et al., 1976); sulfide content was determined using iodometric titration method (Pawlak and Pawlak, 1999) with a titrator. All soil data were expressed based on their oven-dry weight (105°C).

### 2.3 DNA extraction and sequencing

DNA of microbes in sediment was extracted from about 0.5 g (wet weight) of sediments using Fast DNA^®^ SPIN Kit for Soil Kits (MP Biomedicals, California, United States) according to the manufacturer’s specifications. Microbial DNA on polycarbonate filters was extracted using the phenol-chloroform-isoamyl alcohol method (Nercessian et al., 2005). The microbes attached to seagrass leaves were rinsed off with 1% sterile NaCl solution, and then the microbial DNA was extracted using Fast DNA^®^ SPIN Kit for Soil Kits (MP Biomedicals, Irvine, United States). The purity and quality of the DNA was tested using Thermo Scientific NanoDrop 2000c (Thermo Scientific, Waltham, United States). The DNA was stored at −20°C for further analysis. Prokaryotic V3–V4 hypervariable regions in 16S rRNA genes were amplified using universal primers 515F (5′-GTGYCAGCMGCCGCGGTAA-3′) and 806R (5′-GGACTACNVGGGTWTCTAAT-3′) (Pichler et al., 2018). The amplicon library was sequenced using the Illumina Nova 6000 platform to obtain paired-end sequences (Guangdong Magigene Biotechnology Co., Ltd. Guangzhou, China).

### 2.4 Sequences processing

The obtained raw paired-end sequences were analyzed using the QIIME version 1.9.1 pipeline (Caporaso et al., 2010). Briefly, the raw sequences were merged into full-length amplicon sequences using Usearch fastq_mergepairs (V10, http://www.drive5.com/usearch/) (Edgar and Flyvbjerg, 2015). The above-processed clean sequences were clustered into operational taxonomic units (OTUs) with a cut-off 0.03 using the UPARSE 11 pipeline (Edgar, 2013), and chimera checking was performed using the UCHIME algorithm (Edgar et al., 2011). The Ribosomal Database Project (RDP) classifier was used to assign OTUs to taxonomic groups based on the SILVA database (Quast et al., 2012) with a minimum confidence of 0.8. To obtain a final OTU matrix that contains the valid read counts of each OTU in each sample, the OTUs that were assigned to chloroplasts or mitochondria, as well as those were not assigned to any kingdom, were discarded.

### 2.5 Microbial community analysis

The number of prokaryotic sequences varied from 41,399 to 103,425 (average 76,668) per sample. To normalize the data, the sequences were rarefied and subsampled to the minimum number in all samples (Zhang et al., 2016). We were interested in assessing whether significant differences existed in α-diversity of the microbial communities between different habitats (sediment, phyllosphere, and seawater), lagoons (XC Bay vs. LA Bay) and levels of eutrophication (heavy pollution-site2 vs. less pollution-site1). For this purpose, we calculated the ACE, Chao1, Shannon2 (log base 2), and Simpson diversity indices based on the OTUs matrices. Two-way ANOVAs was performed to identify the main and interactive effects of the lagoons and sampling sites (eutrophication) on the α-diversity of the microbial communities, after which *post hoc* Tukey’s HSD test was applied to test the pairwise group differences. The normality of each index was determined using the Shapiro–Wilk test (Chao, *p* > 0.05; ACE, *p* > 0.05; Shannon, *p* > 0.05; Simpson, *p* > 0.05) (Royston, 1992).

To visualize the community dissimilarity between sites, principal coordinate analysis (PCoA) were applied based on Bray–Curtis dissimilarities of the OTU matrices using vegan package (Dixon, 2003) in R. A one-way permutational multivariate ANOVA (PERMANOVA) (Anderson, 2005) was applied to identify the differences of microbial community among the three habitats and among the four sampling sites of each habitat based on Bray–Curtis dissimilarities of the OTU matrices. The overall compositional differences between the lagoons and sites of each habitat were tested using two-way PERMANOVA. The permutation test of multivariate homogeneity of group dispersions was applied to evaluate the effect of group dispersion on the PERMANOVA results (Anderson, 2006). The linear discriminant analysis effect size (LEfSe), based on the relative abundance of OTUs at the genus level, was applied to identify the groups that display significant differences in relative abundance between XC and LA lagoons, and between site1 and site2 within each habitat (Segata et al., 2011). The bacterial functions were predicted by FAPROTAX based on the OTU matrices (Louca et al., 2016).

### 2.6 Correlation analysis

The correlations between microbial community compositions and physicochemical parameters were examined using Mantel test, with dissimilarity matrices of microbial communities based on Bray–Curtis distances between samples. Pearson tests were run to determine the correlations between the physicochemical parameters and α-diversities. The analyses were performed and the results were visualized by using the packages linkET (Huang, 2021) and dplyr (Wickham et al., 2021) in R. The Hmisc package in R was used to examine the Pearson correlations between OTUs and environmental variables (Harrell and Harrell, 2019). Only the top 100 OTUs in total relative abundance across all samples from each habitat are used for analysis. The interactive platform gephi was used to visualize networks (Bastian et al., 2009) with only correlations with absolute value of *r* > 0.6 and *p* < 0.05 were retained as valid co-occurrence events.

To access the complexity of the microbial community within the seagrass ecosystems, a co-occurrence network consisting of all samples at each site was constructed. To avoid potential spurious correlations, the top 500 dominant OTUs in each site were selected. The correlation among microbial OTUs was accessed based on Pearson’s correlation, and correlation coefficients >0.8 with a corresponding of *p*-value <0.01 were retained to generate the network. All networks were constructed using ggClusterNet package in R (Wen et al., 2022), and visualized in *Fruchterman–Reingold* layout using the interactive Gephi platform.^1^ To describe the topology and compare the complexity of each network, we calculated a set of metrics, including nodes, edges, average degree, network diameter, average path length, graph density, modularity, and average clustering coefficient, in Gephi. A higher number of nodes and edges, average degree, average clustering coefficient, and graph density, along with lower average path lengths, indicating greater potential complexity within networks (Wang et al., 2023).

## 3 Results

### 3.1 Physicochemical parameters

In seawater, the temperature (29.5–33.3°C), salinity (33.3–34.0), and pH (7.85–7.89) showed slight variations among the sites XC1, XC2, LA1, and LA2 (Table 1). The COD and the concentration of Chl*a* and LP were several micrograms per liter at the four sites, with higher levels observed at site2 (XC2 or LA2) than at site1 (XC1 or LA1) in each lagoon. DO was lower at site2 compared to site1 in each lagoon. The TIN concentrations were high (7.87–12.18 μM) across all sites, with the concentrations of NH_4_^+^, NO_3_^−^, and NO_2_^−^ were higher at XC1 than at XC2, but lower at LA1 than at LA2. The higher COD and LP content but lower inorganic nitrogen content at XC2 compared to XC1 might be because the higher concentrations of organic carbon and phosphorus nutrients stimulated the assimilation of inorganic nitrogen by heterotrophic microorganisms, thereby decreasing its concentration (Deng et al., 2021). In sediment, the content of all tested nutrients, including OC, sulfide, TP, and TN, especially the sulfide, were higher at site2 than at site1 in each lagoon (Table 1). These results indicate that the site2 (XC2 or LA2) was more eutrophic than site1 (XC1 or LA1) in each lagoon, and the XC lagoon was relative more eutrophic than the LA lagoon.

**Table 1:** Physicochemical parameters of the seawater and sediment at sampling sites.

Habitats	Parameters	Sampling sites			
		XC1	XC2	LA1	LA2
Seawater	Temperature (°C)	31.1	32.2	33.3	29.5
	Salinity (‰)	33.4	33.3	34.0	33.6
	pH	7.89	7.86	7.86	7.85
	Chl*a* (mg L^−1^)	1.40	1.81	1.40	2.08
	DO (mg L^−1^)	6.32	6.17	7.18	6.42
	COD (mg L^−1^)	1.26	3.89	1.98	2.63
	NH_4_^+^ (μM)	2.861	2.385	1.910	2.236
	NO_3_^−^ (μM)	9.272	7.016	5.923	8.179
	NO_2_^−^ (μM)	0.050	0.035	0.038	0.108
	TIN (μM)	12.182	9.437	7.871	10.523
	LP (μM)	0.233	0.378	0.061	0.474
Sediment	OC (%)	1.35	2.03	2.64	3.15
	Sulfide (μmol g^−1^)	0.161	0.844	0.371	0.697
	TP (μmol g^−1^)	12.258	16.419	7.258	12.774
	TN (μmol g^−1^)	30.643	32.571	29.143	32.000

DO, dissolved oxygen; COD, chemical oxygen demand; TIN, total inorganic nitrogen; LP, labile phosphorus; OC, organic carbon content; TP, total phosphorus; TN, total nitrogen.

### 3.2 α-diversity of microbial community

**Figure 2:**
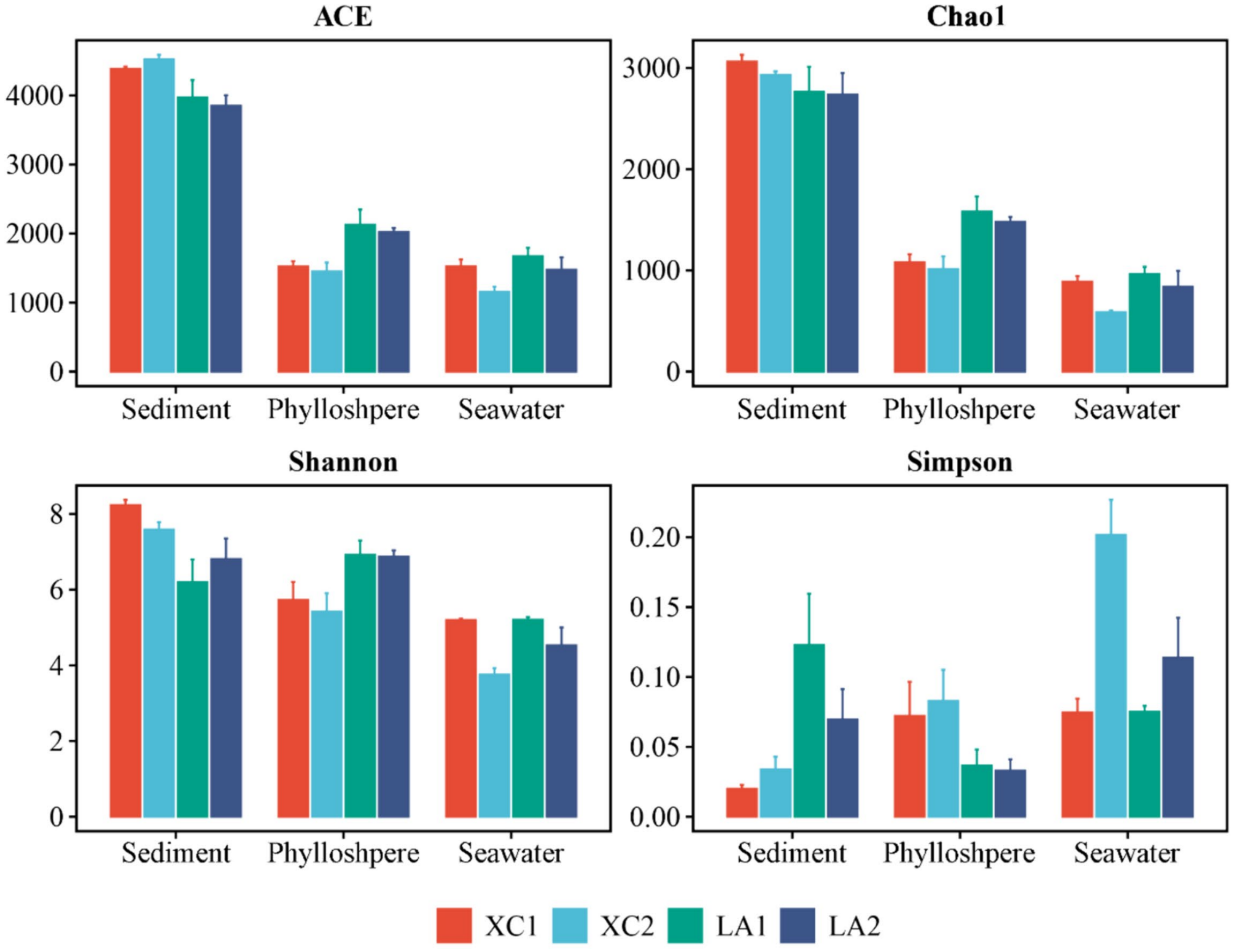
The values of α-diversity index ACE, Chao1, Shannon, and Simpson of sediment, phyllosphere, and seawater microbial communities are shown as bar plot. The bar color represents different sites.

The α-diversity indices, including ACE, Chao1, and Shannon, showed a successive and significant decrease in microbial diversity from sediment to seagrass leaves to seawater (Supplementary Figure S1). The Simpson index also showed that microbial diversity in sediment and on seagrass leaves was significantly higher than in seawater (Supplementary Figure S1). For each habitat, the ACE (*p* < 0.001), Shannon (*p* < 0.005), and Simpson (*p* < 0.005) indices indicated that sediment microbial diversity in the XC lagoon was higher than in the LA lagoon. However, all α-diversity indexes (ACE, *p* < 0.01; Chao1, *p* < 0.01; Shannon, *p* < 0.05; Simpson, *p* < 0.05) suggested that the phyllosphere microbial diversity in the XC lagoon was lower than those in the LA lagoon (Figure 2 and Supplementary Table S1). Moreover, the α-diversity indices (Chao1, *p* < 0.01; Shannon, *p* < 0.05; Simpson, *p* < 0.05) mostly indicated that seawater microbial diversity was higher at the less eutrophic site compared to the highly eutrophic site in both lagoons (Figure 2 and Supplementary Table S1).

### 3.3 Community composition of prokaryotic 16S rRNA gene among the habitats

**Figure 3:**
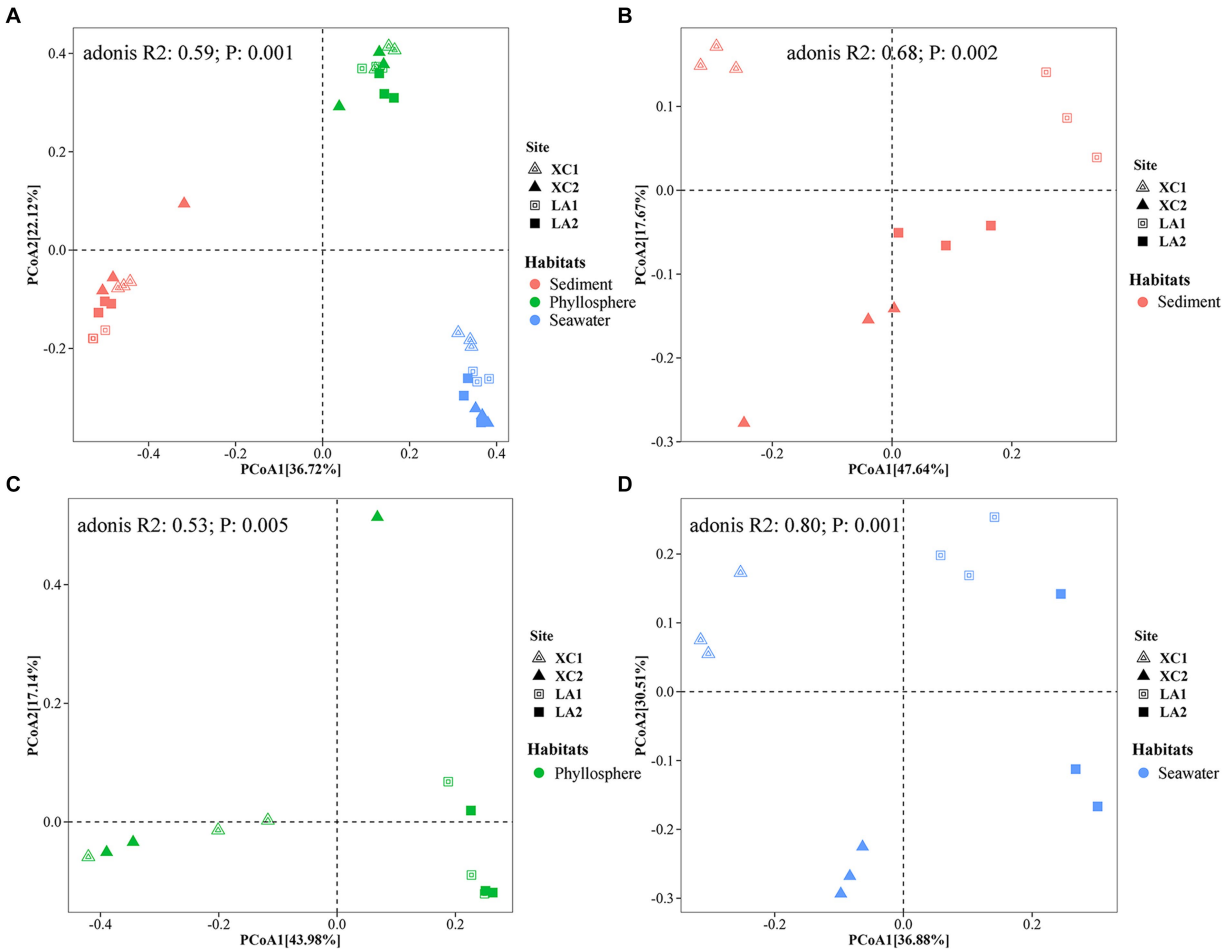
Principal coordinate analysis (PCoA) revealed the differences of the prokaryotic community composition among the habitats of sediment (red), seagrass leaves (green), and seawater (blue) **(A)**, and among the four sampling sites in sediment **(B)**, on seagrass leaves **(C)**, and in seawater **(D)**, respectively.

**Figure 4:**
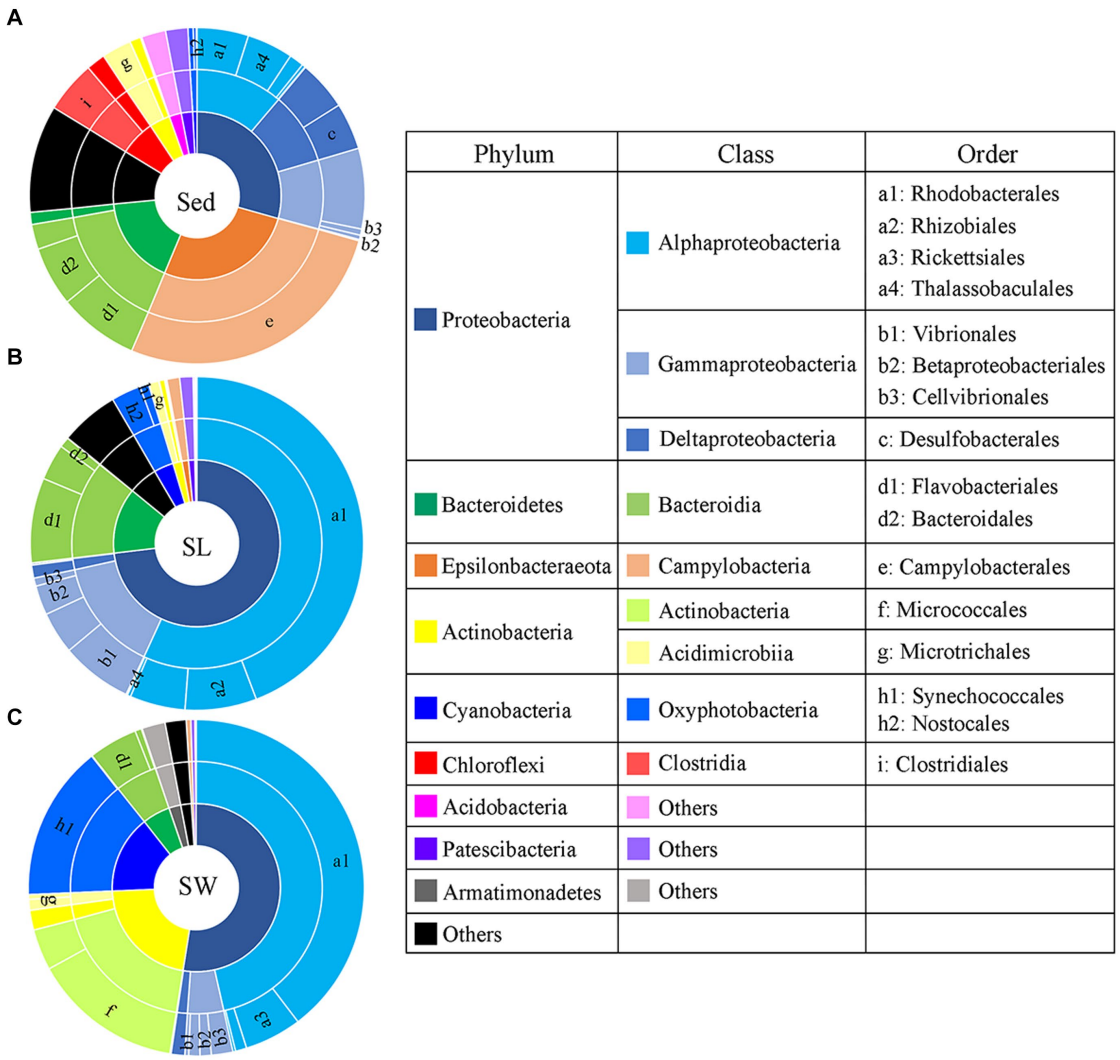
Taxonomic composition of microbial communities from the **(A)** sediment (Sed), **(B)** seagrass leaves (SL), and **(C)** seawater (SW). The pie area represents the average relative abundance of taxa in each habitat. The unlabeled area in family level are marked as the others with relative abundance <2%.

PCoA showed a clear distinction of microbial community composition among the sediment, seagrass leaves, and seawater habitats (Figure 3A). The average microbial relative abundance at levels from phylum to family in the three habitats is shown in Figure 4. *Alphaproteobacteria*, *Gammaproteobacteria*, and *Deltaproteobacteria* were the three most dominant class in Proteobacteria, with *Deltaproteobacteria* being abundant only in sediment (9.3%), and *Alphaproteobacteria* having the highest relative abundance on seagrass leaves (57.0%) and in seawater (46.5%). *Gammaproteobacteria* was most abundant on seagrass leaves (14.7%) compared to sediment (8.8%) and seawater (4.6%). *Vibrionales* within *Gammaproteobacteria* was most abundant on seagrass leaves. The *Deltaproteobacteria* in sediment was mainly composed of *Desulfobacterales*. The relative abundance of the class *Bacteroidia* gradually decreased from sediment (15.9%) to seagrass leaves (12.7%) to seawater (5.4%). Within the phylum *Actinobacteria*, the class *Actinobacteria* (mainly *Micrococcales*) was detected only abundant in seawater (14.5%), while *Acidimicrobiia* was detected with the relative abundance ranging from 1 to 2.9% in all three habitats. *Campylobacteria* (phylum *Epsilonbacteraeota*) was the most abundant class in sediment (27.0%) and had a minor proportion on seagrass leaves (1.3%). The phyla *Chloroflexi* (5.0%) and *Acidobacteria* (2.3%) were exclusively abundant in sediment. *Armatimonadetes* was exclusively abundant in seawater (2.3%).

### 3.4 Comparison of prokaryotic community structure among sites in each habitat

PCoA analyses indicated that microbial community structure differed significantly among the four sampling sites (Figures 3B–D). Further two-way PERMANOVA analyses (Supplementary Table S2) showed that lagoons (XC vs. LA) played a major role in determining sediment microbial composition (*F* = 9.2, *p* = 0.001) compared to sampling sites (site1 vs. site2) (*F* = 3.8, *p* = 0.022; Figure 3B). In contrast, both lagoon (*F* = 13.9, *p* = 0.001) and sampling sites (*F* = 11.4, *p* = 0.001; Figure 3D) played similar roles in determining seawater microbial composition. The combined effect of lagoons and sampling sites was also statistically significant for both sediment (*F* = 4.3, *p* = 0.009) and phyllosphere (*F* = 5.9, *p* = 0.002) microbial community. For seagrass leaves, microbial composition was significantly affected by lagoons (*F* = 6.3, *p* = 0.001) but not by sampling sites (*F* = 1.3, *p* = 0.213; Figure 3C).

**Figure 5:**
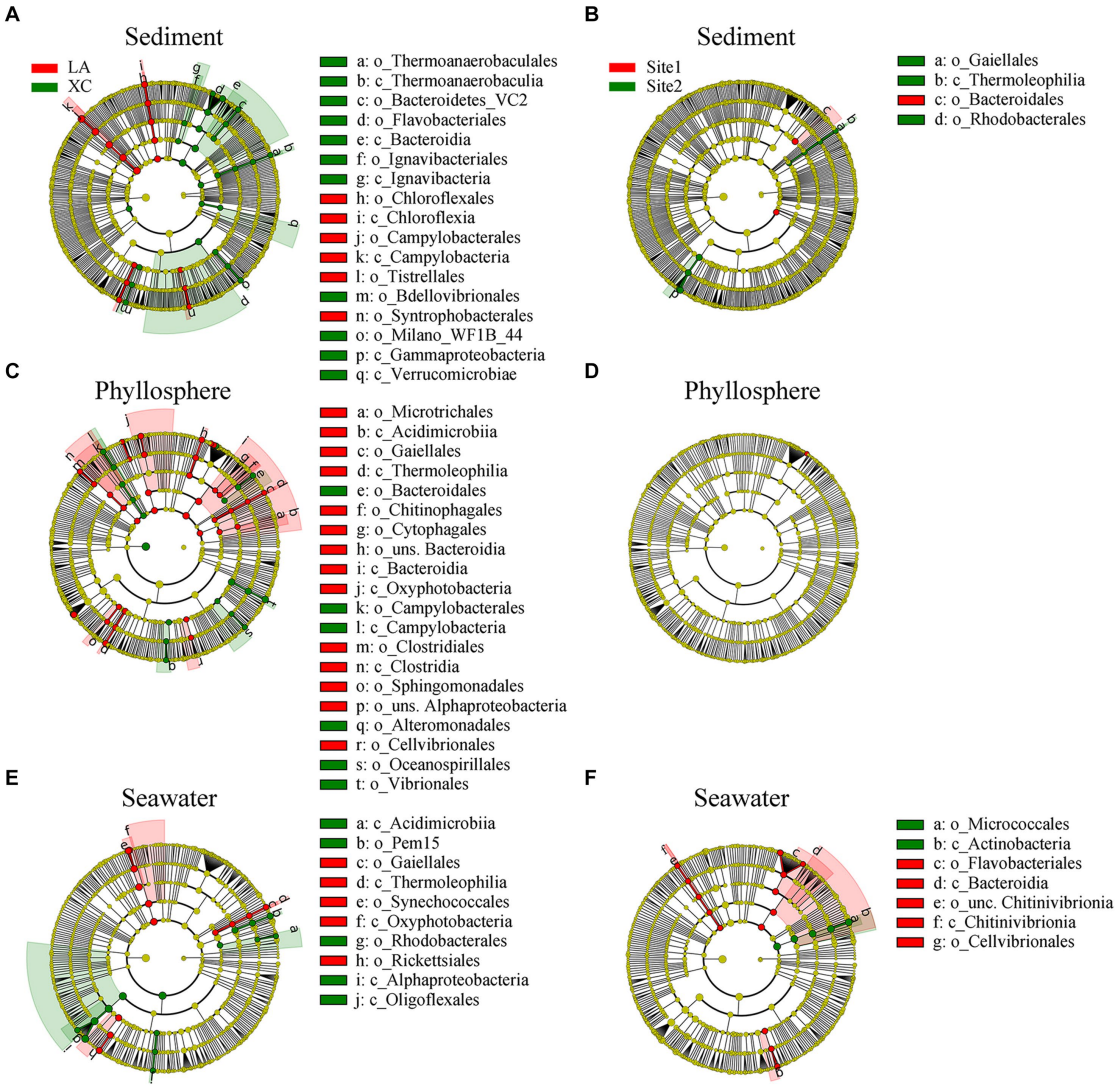
Relative abundance of sediment **(A,B)**, phyllosphere **(C,D)**, and seawater **(E,F)** prokaryotic taxa significantly differentiated between XC and LA **(A,C,E)** and between site1 and site2 **(B,D,F)**, identified by linear discriminant analysis effect size (LEfSe). Only lineages with linear discriminate analysis (LDA) values >3.5 are displayed. The diameter of each small solid circle is proportional to abundance of the given taxon. The multiclass analysis strategy was all-against-all (more strict). Large circles indicate phylogenetic levels (from domain to genus) in reverse order. Classes and orders are labeled.

LEfSe results showed significant differences in the relative abundance of microbial groups (including class and order) between the XC and LA lagoons: 17 groups in sediment, 20 groups in the phyllosphere, and 10 groups in seawater (Figures 5A,C,E). However, there were only 4 groups in sediment, 0 groups in the phyllosphere, and 7 groups in seawater showing significant differences between highly and less eutrophic sites (Figures 5B,D,F). On seagrass leaves, *Campylobacterales*, *Bacteroidales*, *Oceanospirillales*, and *Vibrionales* were enriched in XC, whereas *Microtrichales*, *Bacteroidia*, and *Clostridiales* were enriched in LA (Figure 5C).

### 3.5 Correlation between microbial community and physicochemical parameters

**Figure 6:**
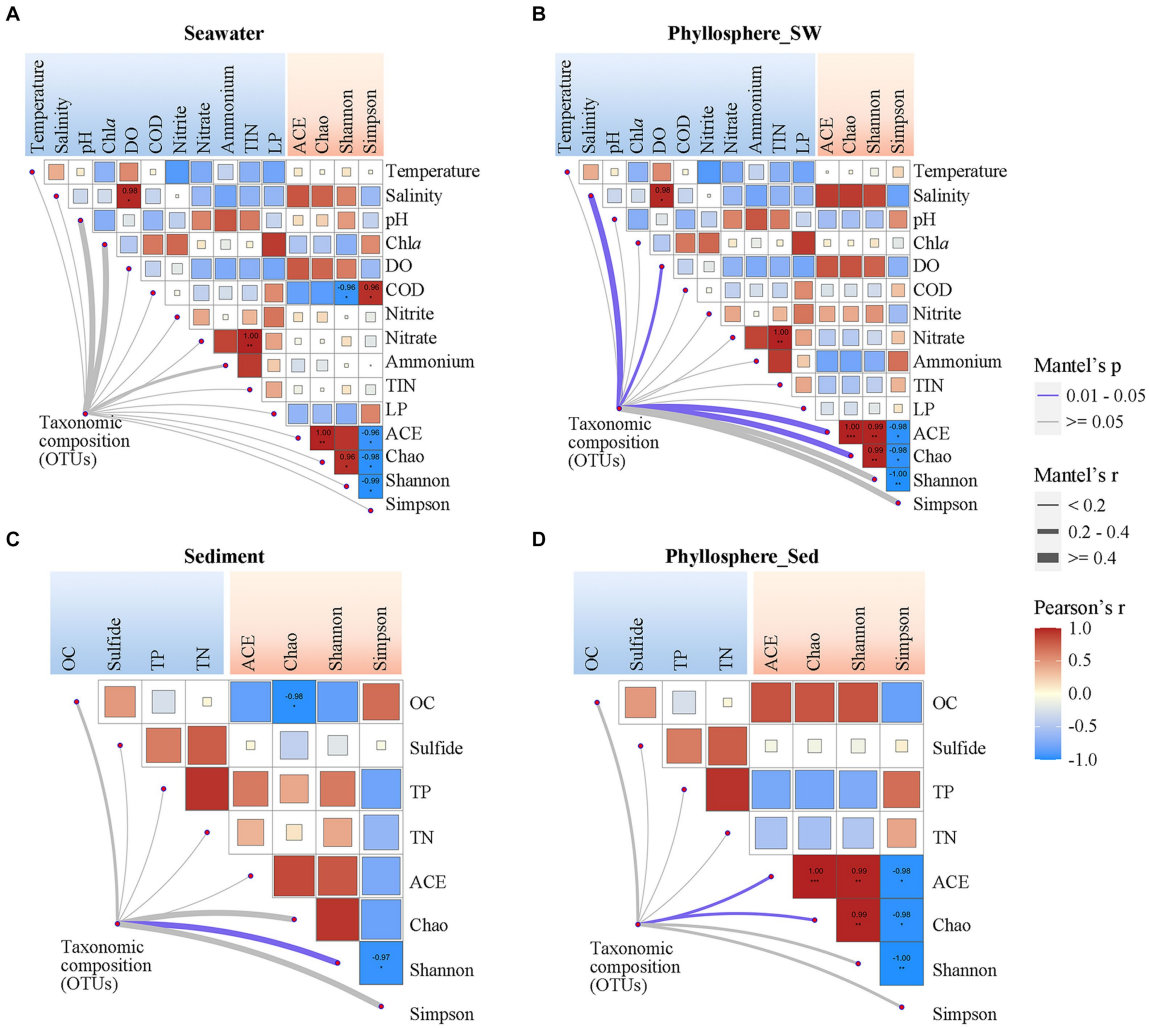
Correlations of microbial community compositions and α-diversity (based on OTUs) with geochemical parameters of **(A)** seawater and **(C)** sediment, and the correlations of phyllosphere microbial compositions and α-diversity with geochemical parameters of **(B)** seawater and **(D)** sediment. The color of lines indicates the *p*-value of the Mantel test; the width of color lines indicates the *r*-value. Asterisks indicate Pearson’s *p* < 0.05 and the color bar is based on Pearson’s correlation coefficients. DO, dissolved oxygen; COD, chemical oxygen demand; TIN, total inorganic nitrogen; LP, labile phosphorus; OC, organic carbon; TP, total phosphorus; TN, total nitrogen.

Pearson correlation analysis showed that seawater and phyllosphere microbial α-diversity exhibited various correlations with seawater physicochemical parameters (Figures 6A,B). For the seawater microbial community, seawater salinity and DO were positively correlated with diversity, while COD, Chl*a* and LP were negatively correlated with diversity, and nitrogen nutrients showed no obvious correlation with diversity (Figure 6A). For the phyllosphere microbial community, seawater salinity and DO were also strongly correlated with diversity, but the correlations of COD, Chl*a*, and LP with diversity were weakened; however, nitrogen nutrients became have some weak correlations with diversity: NO_2_^−^ was positively correlated, and NO_3_^−^ and NH_4_^+^ were negatively correlated (Figure 6B). Regarding sediment physicochemical parameters, OC had a negative correlation, while TP and TN showed a positive correlation with sediment microbial diversity. These correlations were reversed for phyllosphere microbial diversity, where OC showed a positive correlation, and TP and TN showed negative correlations (Figures 6C,D). Sulfide showed no obvious correlation with either sediment or phyllosphere microbial diversity (Figures 6C,D).

Beside seagrass salinity and DO being significantly linked with phyllosphere microbial composition, the physicochemical parameters generally showed no significant correlation with microbial compositions (Figure 6). The co-occurrence network showed that microbial taxa (OTUs) were generally connected with only one or two physicochemical parameters, except that many OTUs of phyllosphere *Rhodobacterales* connected with two or more seawater physicochemical parameters (Supplementary Figure S2). Notably, among the seawater physicochemical parameters, NO_2_^−^ had the highest number of links with seawater and phyllosphere microbial taxa. For sediment physicochemical parameters, OC had the highest number of links with phyllosphere microbial taxa.

### 3.6 Microbial community analysis by using co-occurrence networks and ternary plot

**Figure 7:**
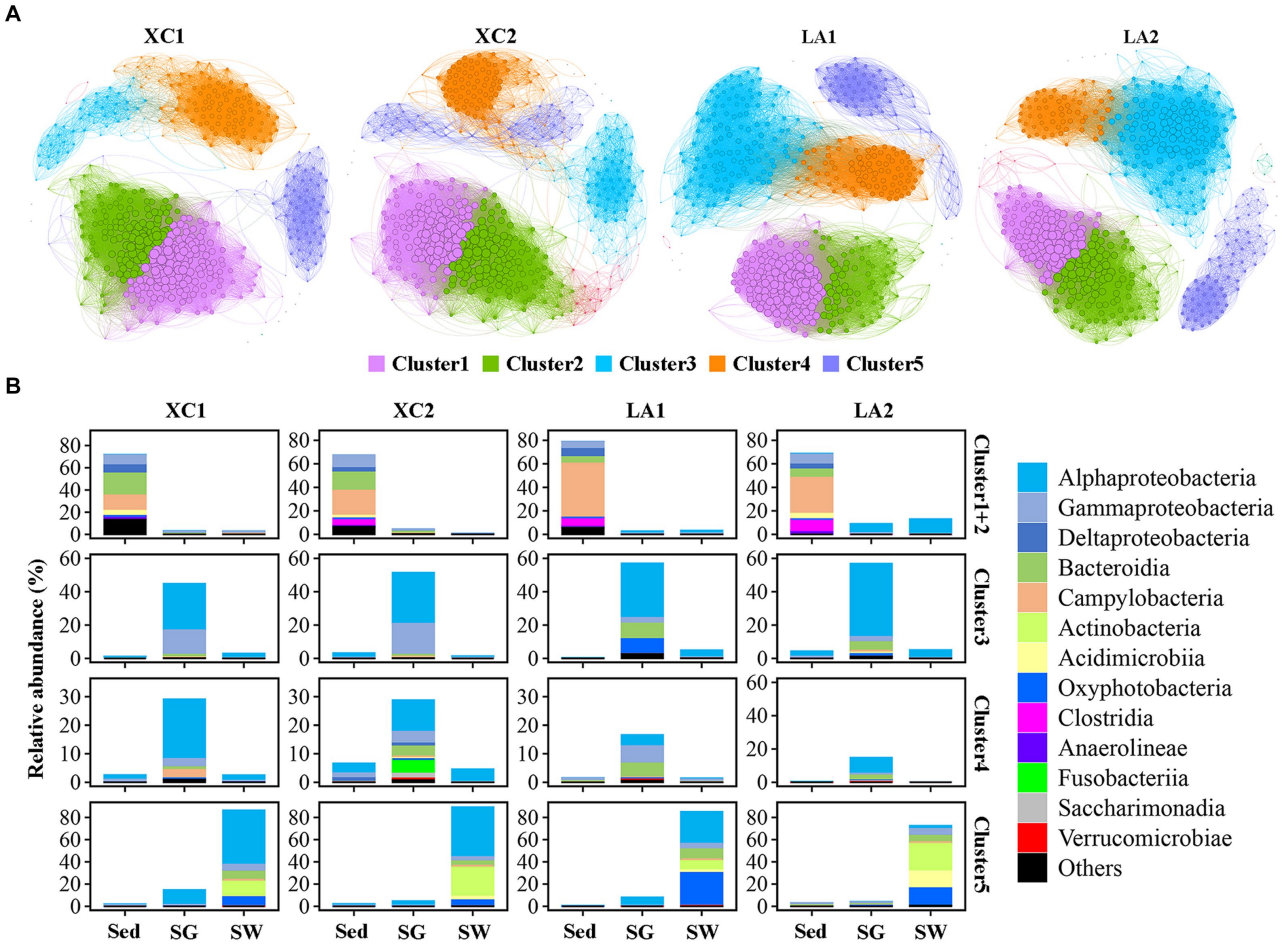
**(A)** Co-occurrence network of the sediment, phylloshpere, and seawater microbial community at each site. Only Pearson’s correlation coefficient (*r* > 0.8 or *r* < 0.8 significant at *p* < 0.01) is shown. The nodes and edges are colored according to cluster. Node size is proportional to the average degree of each OTU, and edge thickness is proportional to the weight of each correlation. **(B)** Bar plot shows the composition of the clusters in class level and their total relative abundance in each habitat at each site.

The topological features of the network varied considerably between the two lagoons and between the highly and less eutrophic sites (Figure 7A and Supplementary Table S3). Compared to the highly eutrophic site in each lagoon, microbial community at the less eutrophic site had a more complex network with higher edges, a higher average degree, density and average clustering coefficient, and a lower average path length and diameter. Five dominant ecological clusters were identified across the four sites (Figure 7A). Members in Cluster 1 and Cluster 2 accounted for the highest relative abundance in sediment (> 67%), Cluster 3 and Cluster 4 were dominated by taxa from seagrass leaves (>72%), and Cluster 5 was dominated by taxa from seawater (>73%) (Figure 7B). Among these clusters, Cluster 1 and Cluster 2 had the highest average degree, and Cluster 5 had the lowest average degree (Supplementary Table S4).

While the average degree of the sediment clusters (Clusters 1 and 2), seawater cluster (Cluster 5), and the phylloshpere Cluster 4 remained relatively stable across the four sites, the average degree of the phylloshpere Cluster 3 decreased greatly from lagoon LA to XC (Figure 7 and Supplementary Table S4). In addition, the percentages of the number of links (edges) between Cluster 1 and Cluster 2 relative to the total edges within these clusters were not higher in the XC lagoon (15.2%–17.2%) compared to the LA lagoon (10.7%–15.2%), whereas those values between Clusters 3 and 4 were much lower (0.3%–1.2%) in XC than in LA (3.2%–3.3%) (Figure 7 and Supplementary Table S5). Additionally, the links between Cluster 3 and Cluster 4 were much weaker at the highly eutrophic site (0.3%) compared to the less eutrophic site (1.2%) in XC, while those values were similar between the highly and less eutrophic sites in the LA (Supplementary Table S5).

**Figure 8:**
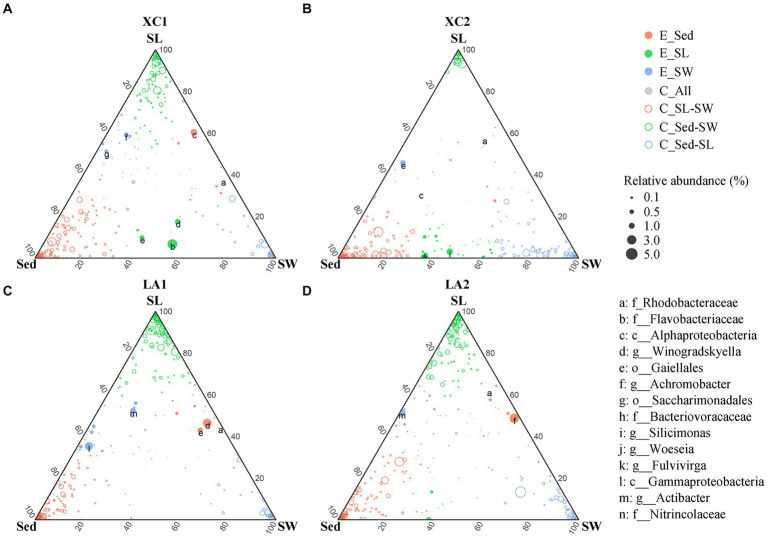
Comparison of microbial composition from the habitats of sediment (Sed), seagrass leaves (SL), and seawater (SW) at **(A)** XC1, **(B)** XC2, **(C)** LA1, and **(D)** LA2 sites. Circle size represents the highest relative abundance of each genus among the three habitats at each site. If the difference in relative abundance of a genus was less than two-fold across the three habitats, it was noted as a core connector (C_All, gray circle). If the relative abundance of a genus in one habitat was at least two-fold higher than its abundance in the remaining two habitats, the genus was noted as an enriched (E.) genus (open circles) in that habitat. The remaining genera with higher relative abundance in two habitats compared to the other habitat were classified as “connector (C)” (closed color circles) between those two habitats. The relative abundance is the average relative abundance of the three parallels in each habitat of each site.

Given the large variation in the relative abundance of each OTU among the three habitats and strict criteria (*r* > 0.8, *p* < 0.01) used for building co-occurrence networks, no connection was observed between clusters dominated by taxa from different habitats in the co-occurrence networks (Figure 7). Therefore, we also used a ternary plot to access the interconnections of microbial groups among the three habitats at each site (Figure 8). The results indicate that most genera were enriched in one of the three habitats (E_Sed, E_SG, and E_SW), with no or few genera sharing similar relative abundance across all three habitats (C_All) at each site. At site XC1, the majority of genera were located near or on the axis SG-Sed, with the fewest genera near or on the Sed-SW axis (Figure 8A). Conversely, at site XC2, most genera were located near or on the Sed-SW axis (Figure 8B). Similarly, the total relative abundance of the connecters between seagrass leaves and sediment (Sed-SG) and between seagrass leaves and seawater (SW-SG) was higher at XC1 than at XC2 (Supplementary Figure S3). However, those values did not differ obviously in the LA lagoon (Supplementary Figure S3).

### 3.7 Functional prediction of the microbial community

**Figure 9:**
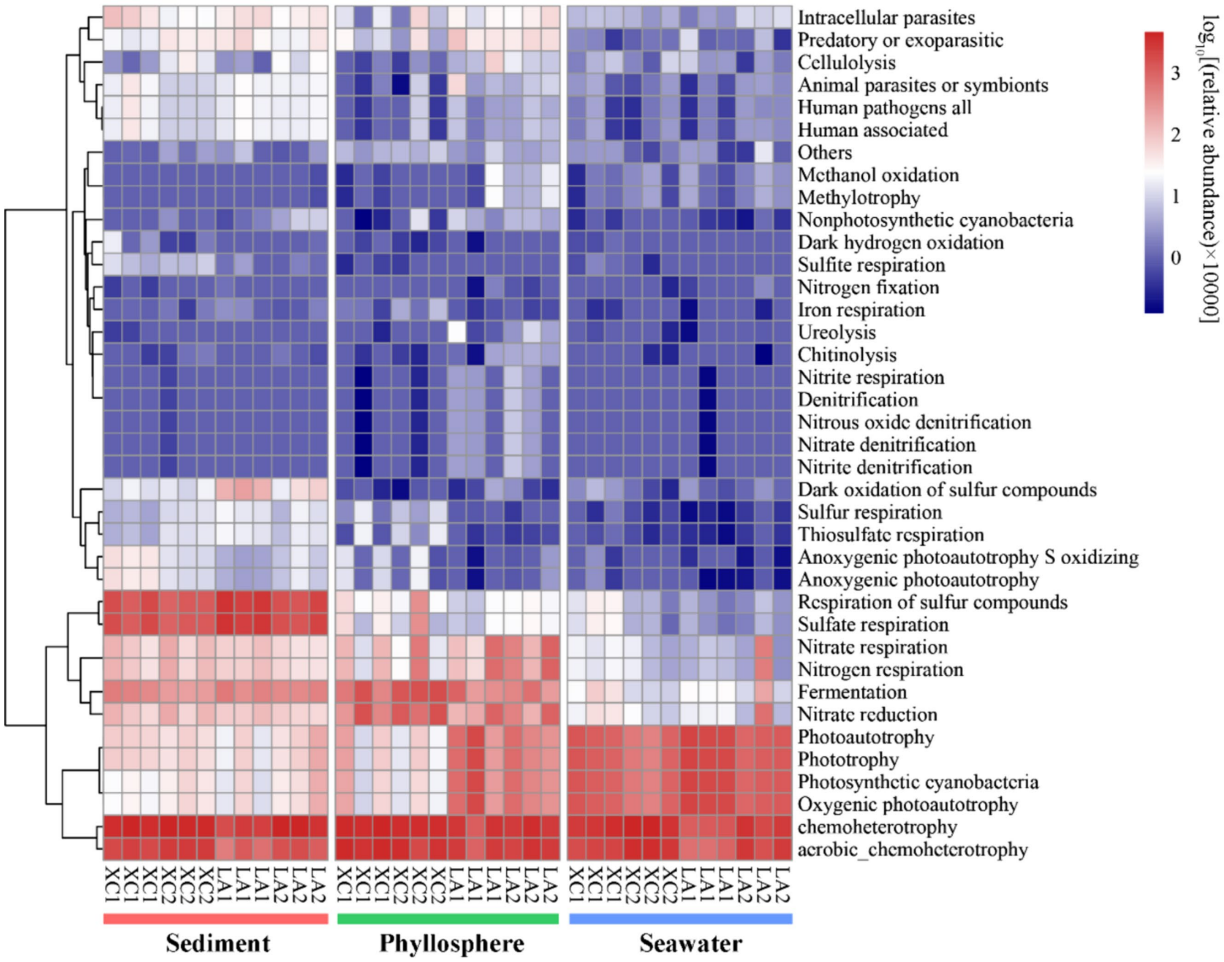
The functional composition of microbial community was predicted by using FAPROTAX. Heatmap represent the relative abundance of functional groups of prokaryotic communities.

Only 2,735 out of 16,212 OTUs (16.9%) could be assigned to at least one function by using FAPROTAX. Chemoheterotrophy/aerobic chemoheterotrophy were the most abundant functions across all three habitats (Figure 9). Sulfate respiration was the second most abundant function in sediment; other functions related to sulfur metabolism were also more abundant in sediment than on seagrass leaves and in seawater. Functions related to nitrogen metabolism (including nitrate respiration, denitrification, nitrogen fixation), as well as methanol oxidation, methylotrophy, chitinolysis, cellulolysis, and ureolysis, were commonly most abundant on LA seagrass leaves. In contrast, nitrate reduction and fermentation were commonly most abundant on XC seagrass leaves. Additionally, functions related to photoautotrophy had a higher relative abundance on LA seagrass leaves compared to XC seagrass leaves.

## 4 Discussion

### 4.1 Differences of sediment, phyllosphere, and seawater microbial community structure within seagrass ecosystem

Previous investigations of microbial communities in coastal ecosystem have indicated that microbial diversity was much higher in sediment than in seawater, with distinct microbial composition observed between these two environments (Won et al., 2017; Cleary et al., 2018; Ul-Hasan et al., 2019). However, these studies did not specifically focus on the phyllosphere microbial community. Our results regarding sediment and seawater microbial diversity in line with these findings and further identify that phyllosphere microbial diversity is significantly higher than in seawater and significantly lower than in sediment (Supplementary Figure S1). The microbial community composition also varied obviously among the three habitats (Figures 3A, 4). For example, the *Desulfobacterales*, which comprises anaerobic sulfate-reducing microorganisms (Burow et al., 2014; Cúcio et al., 2016; Dyksma et al., 2018), and *Sulfurovum*, which primarily consists of sulfur-oxidizing chemoautotrophs (Inagaki et al., 2004; Giovannelli et al., 2016; Xie et al., 2021), were most abundant in sediment (Figure 9).

High abundance of both sulfur reducers and oxidizers have been reported in the sediment of various seagrass ecosystem (Zhang et al., 2020; Rabbani et al., 2021; Mohapatra et al., 2022). However, in non-seagrass coastal sediment, only sulfur reducers are typically found in high abundance (Cleary et al., 2018; Ul-Hasan et al., 2019; Chen et al., 2020). Previous studies have shown that high concentration of nutrients and organic matter stimulated the abundance of *Campylobacterales* in non-seagrass coastal sediment (Aires et al., 2019; Salem et al., 2019). Our co-occurrence network analysis also showed that the most abundant OUT of *Campylobacterales* positively connected with organic carbon content (OC) within sediment (Supplementary Figure S2C). These findings likely explain the high abundance of sulfur oxidizers in seagrass sediments: seagrass provides abundant organic matter to the sediments, and sulfur reducers can utilize organic matter as electron donors to produce reduced sulfide (Wu et al., 2021), which in turn stimulates the growth of sulfur oxidizers. Since reduced sulfur compounds, especially hydrogen sulfide, are known phytotoxins in eutrophic sediment (Goodman et al., 1995; Lamers et al., 2012), their oxidation by the abundant *Campylobacterales* generated a positive feedback loop of the organic matter provided by seagrass. This process helps mitigate the toxic effects of hydrogen sulfide and promotes seagrass adaptation in the two lagoons (Zhang et al., 2020).

Another function that notably differed among the three habitats was organic matter degradation. Taxa such as *Clostridiales*, *Thalassobaculales*, and *Bacteroidales*, which were predominantly abundant in sediment, play important roles in degrading recalcitrant organic matters, such as seagrass cell wall polysaccharides and humic-rich organic matter generated through the biochemical transformations of dead seagrass (Riviere et al., 2009; van der Lelie et al., 2012; Vanwonterghem et al., 2014; Aires et al., 2019; Grevesse et al., 2022). In contrast, the order *Rhodobacterales*, which was the most abundant group in seawater and on seagrass leaves, is well-known for its rapid response to low-molecular-weight organic matters (Taylor et al., 2014; Varela et al., 2020; Pontiller et al., 2022). *Micrococcales*, known for its importance in degrading refractory organic matters such as cellulose, lignin, and polyaromatic hydrocarbons (Liu et al., 2020), was the second most abundant taxon in seawater (Figure 4). Thus, *Micrococcales* in eutrophic seawater of seagrass ecosystem is likely an important degrader of organic matter derived from dead seagrass biomass.

As seagrass is a key species in seagrass ecosystem, it is crucial to highlight the functions that enriched in the phyllosphere microbial community. Several important functions, such as denitrification, nitrogen fixation, urelysis, methylotrophy, chitinolysis, and cellulolysis, were observed to be enriched on seagrass leaves (Figure 9). This is reasonable because cellulose, methanol, and chitin are exudates released by seagrass leaves (Venkatachalam et al., 2015; Sanders-Smith et al., 2020), which can obtain inorganic nitrogen with the assistance of microbial nitrogen fixation (Agawin et al., 2016) and the degradation of dissolved organic nitrogen (Tarquinio et al., 2018). While seagrass sediment is well-known as a hotspot for denitrification and nitrogen loss (Hemminga et al., 1991; Garcias-Bonet et al., 2018), phyllosphere denitrification has received comparatively less attention. Our findings suggest that denitrification on seagrass leaves may also serve as a potential pathway for nitrogen loss, thereby balancing coastal anthropogenic nitrogen inputs. Consequently, our results revealed that sediment, seagrass leaves, and seawater in seagrass ecosystem are occupied by distinct microbial groups dedicated to metabolizing various kinds of organic matters and nutrients. This diversity in microbial functions is essential for maintaining the health of seagrass ecosystems.

### 4.2 Differential impact of eutrophication on seagrass microbial communities compared to sediment and seawater microbial communities

High microbial diversity can enhance the stability and resilience of ecosystems, because microbial communities with high diversity are more likely to include species that can adapt to new environments when ecosystems are subjected to external disturbances (Girvan et al., 2004). In the present work, sediment microbial diversity was higher in the relative more eutrophic XC lagoon than in the LA lagoon, while phylloshpere microbial diversity was higher in LA than in XC (Figure 2). This result indicates that the diversity of phyllosphere microbial communities is more susceptible to reduction due to eutrophication compared to the diversity of sediment microbial communities, potentially leading to the decay of seagrass leaves under eutrophic conditions. This inference can be supported by the enrichment of representative *Oceanospirillales* and *Vibrionale*s on seagrass leaves in the relative more eutrophic XC lagoon (Figures 4, 5C). Because, the *Oceanospirillales* and *Vibrionales* were also observed a significant increase in abundance on seaweed due to environmental degradation (Aires et al., 2018), and *Vibrionales* is a representative microorganism that commonly associated with stress and diseased seaweeds, coral, and animals (Meron et al., 2011; Vezzulli et al., 2016; Aires et al., 2018).

We also found that the relative harsher environment in the XC lagoon reduced the relative abundance of phyllosphere functional taxa related to important functions, such as denitrification, while increasing the relative abundance of anaerobic functional taxa involved in processes like fermentation and nitrate reduction (Figure 9). This shift may accelerate seagrass leaf decay and reduce denitrification, exacerbating eutrophication. Additionally, the phyllosphere microbial diversity and compositions between the highly and less eutrophic sites were more similar (Figure 3C) compared to those of seawater microbial compositions (Figures 3D, 5D). This indicates that seagrass leaf plays a pivotal role in shaping the microbial community and maintaining the stability of community structure by releasing exudates such as methanol, cellulose, and phenols (Papazian et al., 2019; Sanders-Smith et al., 2020) under different eutrophic conditions. Another possibility is that the phyllosphere microbial community at the less eutrophic site had been subjected to similar degree of impact by eutrophication as at the highly eutrophic site in each lagoon. The microbial-mediated plant communication theory is suitable to interpret this possibility: the secretion of signaling substances under certain conditions triggers the expression of plant microbiome-shape genes, thus establishing unique plant-associated microbial community; the influenced microbes might, in turn, regulate the plants themselves and other spatially proximate plants via released volatile organic compounds or quorum-sensing signaling molecules (Liu et al., 2023; Lv et al., 2024).

Correlation analysis indicated that COD and OC were the primary factors influencing the seawater and sediment microbial diversity, respectively, and high levels of COD and OC were associated with reduced diversity (Figures 6A,C). This is because a high content of organic carbon can lead to strong selection for specialist hydrocarbon-degrading microorganisms, such as the *Rhodobacterales* in this study, resulting in the disappearance of certain groups of microorganisms (Nogales et al., 2011). Unlike COD, which only influenced seawater microbial diversity, factors such as the salinity and DO, had similar influence on both seawater and phylloshpere microbial diversity (Figures 6A,B). However, this did not mean that organic carbon was not related to phylloshere microbial diversity. Because, the degradation of high levels of organic matter can reduce DO. The lack of an obvious correlation between seawater COD and phylloshere microbial diversity might be because the phylloshere microbial community can be more directly affected by seagrass-derived organic carbon (Ugarelli et al., 2017).

Microbial composition was not correlated with almost all physicochemical parameters (Figure 6). However, in some environments such as terrestrial grasslands and starved seawater macrocosm, microbial composition was showed strong correlation with environmental factors (Zhang et al., 2021; Cornell et al., 2023). Compared with those relative stable environments, the environmental factors in the XC and LA lagoons can be changed by various pathways, including human activities, seagrass, and tidal action, resulting in more complex and variable interactions between the microbial community and environmental factors. The fact that the majority of the top 100 abundant taxa have no correlation with any environmental factors (Supplementary Figure S2) also suggests that the factors influencing their relative abundance varied across different sites. Meanwhile, we still detected some taxa strongly correlated with environmental factors (Supplementary Figure S2), and some of those correlations are known reasonable. For example, *Flavobacteriales*, the well know degrader of high-molecular-weight organic matters (Bernardet, 2015), were positively corelated with COD in seawater (Supplementary Figure S2A), and *Desulfobacterales*, a sulfur reducer, were positively correlated with sulfide in sediment (Supplementary Figure S2C). The most unexpected result is that seawater NO_2_^−^ had the most number of links with various seawater and phyllosphere taxa (Supplementary Figures S2A,B), despite its concentration being much lower than NO_3_^−^ and NH_4_^+^ (Table 1). This work highlights the importance of paying attention to the influence of seawater NO_2_^−^ on seawater and phyllosphere microbial composition.

### 4.3 Significant effects of eutrophication on the complexity of phyllosphere microbial community

The various effects of eutrophication on sediment, phyllosphere, and seawater microbial diversity and composition might influence the microorganisms-mediated flow of energy and elements within the seagrass ecosystem (Konopka, 2009; Ren et al., 2023), thereby impacting the health conditions of the seagrass ecosystem. In ecosystems, exchanges of energy and elements among different species can give rise to complex interactions (Montoya et al., 2006; Yuan et al., 2021), often represented as networks (Pržulj and Malod-Dognin, 2016). It is well-documented that there are links between network complexity and ecosystem functioning and stability (Landi et al., 2018; Yuan et al., 2021; Wang et al., 2023). The co-occurrence network of the integral microbial community from the three habitats at each sampling site revealed network complexity at the highly eutrophic site was lower than at the less eutrophic site in each lagoon and the difference in network properties between the highly and less eutrophic sites was greater in the more eutrophic XC lagoon than in the less eutrophic LA lagoon (Supplementary Table S3). Thus, although the microbial diversity and composition analysis, specifically in sediment and on seagrass leaves, did not find obvious differences between highly eutrophic and less eutrophic sites in each lagoon, the co-occurrence network analysis indicated that heavy eutrophication decreased the complexity of microbial communities and, consequently, the multi-functionality of seagrass leaves (Cheng et al., 2021; Wang et al., 2023).

Moreover, the greater decrease in the connections among phylloshpere microorganisms (Figure 7 and Supplementary Table S4) indicates that phylloshpere microbial complexity may be more susceptible to reduction than sediment and seawater microbial complexity under eutrophic conditions. In addition, there was a greater decrease in internal connections within the phyllosphere microbial community compared to the sediment microbial community under more eutrophic conditions (Figure 7 and Supplementary Table S5). The ternary plot also depicted a similar trend, showing that heavy eutrophication obviously decreased the interaction of phyllosphere microbial communities with sediment and seawater microbial communities, and this trend was more pronounced in the more eutrophic XC lagoon (Figure 8). As microorganisms can communicate with each other by releasing volatile organic compounds or quorum-sensing signaling molecules (Liu et al., 2023), the decrease in internal and external interactions of the phylloshpere microbial community might limit the response of seagrass leaves to environmental changes in sediment, seawater, and on seagrass leaves.

By examining the spatial network dynamics of sediment, phyllospere, and seawater microbial communities within seagrass ecosystem in response to eutrophication, this study provides important insights into the microbial mechanisms by which eutrophication threatens seagrass health. Although environmental changes are known to trigger complex interactive effects on seagrass-associated microbial community structure (Korlević et al., 2021; Vogel et al., 2021; Markovski et al., 2022), it is not clear whether eutrophication has different effects on the microbial community structures in the three habitats. This study documents that eutrophication significantly decreased the complexity and internal interactions of the phyllosphere microbial community more than those of the sediment microbial community, and also reduced the external interaction of the phyllosphere microbial community with sediment and seawater microbial communities. Microbial network complexity is closely associated with the functional structure of microbial communities and ecosystem functional processes (Yuan et al., 2021). Therefore, the phyllosphere microbial community is more susceptible to functional decline due to eutrophication, suggesting that seagrass decline is more likely to originate from the decline in the functions of the phyllosphere microbial communities rather than sediment microbial communities. Furthermore, since networked communities have strong linkages with ecosystem functioning, our work suggests that preserving seagrass phyllosphere microbial network structure should be prioritized over the sediment microbial network structure for future seagrass ecosystem conservation or restoration. In addition, similar to microbial biodiversity, which is dependent on both space and time, network features are also temporally dynamic (Yuan et al., 2021). Thus, future studies on microbial networks within seagrass ecosystem need to integrate the spatial dynamics along with their temporal dynamics for seagrass ecosystem conservation or restoration.

## 5 Conclusion

In summary, through the simultaneous investigation of sediment, phylloshpere, and seawater microbial communities within seagrass meadow ecosystems that suffered different degrees of eutrophication, we assessed the various effects of eutrophication on microbial communities from the three habitats. The results demonstrated that eutrophication had more pronounced effects on the decrease in biodiversity and complexity of the phyllosphere microbial community compared to those of sediment and seawater microbial communities. This can lead to greater functional loss in the phyllosphere microbial community, reducing their ability to effectively assist seagrass leaves in adapting to environmental changes (Cornell et al., 2023). This work contributes to the understanding of the microbial mechanisms underlying the effects of eutrophication on the health of seagrass ecosystems and highlights the importance of focusing on microbial communities on seagrass leaves during seagrass meadow conservation and restoration. High-resolution spatial and time-series monitoring of phyllosphere microbial communities may become a feasible approach to assist in seagrass conservation and restoration.

## Data availability statement

Raw sequencing data of the prokaryotic 16S rRNA gene are available at National Center for Biotechnology Information (NCBI) Sequence Read Archive under BioProject accession number PRJNA1113396 with BioSample accession number SAMN41454142 – SAMN41454177.

## Author contributions

WD: Conceptualization, Data curation, Formal analysis, Funding acquisition, Visualization, Writing – original draft. SuC: Investigation, Writing – original draft. SiC: Data curation, Investigation, Writing – original draft. BX: Investigation, Writing – original draft. ZC: Resources, Writing – original draft. YZ: Conceptualization, Writing – review & editing. BC: Writing – review & editing. GC: Conceptualization, Funding acquisition, Project administration, Supervision, Writing – review & editing.

## Footnotes

^1^https://gephi.org
