# Supplementary material for: Impacts of eutrophication on microbial community structure in sediment, seawater, and phyllosphere of seagrass ecosystems
Source: Front Microbiol. 2024 Aug 14;15:1449545. doi: 10.3389/fmicb.2024.1449545 (PMC11350616; doi:10.3389/fmicb.2024.1449545)
Supplement: Supplementary file 1 [file Data_Sheet_1.docx]

**Various effects of eutrophication on sediment, phyllosphere, and seawater microbial community structure in Xincun Seagrass Ecosystem, Hainan, China**

Wenchao Deng^1,2^, Shunyang Chen^1,2,5^, Shiquan Chen^3^, Bingpeng Xing^1,2^, Zhuhua Chan^1^, Yao Zhang^4*^, Bin Chen^1,2,5^, Guangcheng Chen^1,2,5^[[1]](#footnote-1)^*^

^1^ Third Institute of Oceanography, Ministry of Natural Resources, Xiamen, China

^2^ Observation and Research Station of Coastal Wetland Ecosystem in Beibu Gulf, Ministry of Natural Resources, Beihai, China

^3^ Hainan Academy of Ocean and Fisheries Sciences, Haikou, China

^4^ State Key Laboratory of Marine Environmental Science and College of Ocean and Earth Sciences, Xiamen University, Xiamen, China

^5^ Key Laboratory of Marine Ecological Conservation and Restoration, Ministry of Natural Resources, Xiamen, China


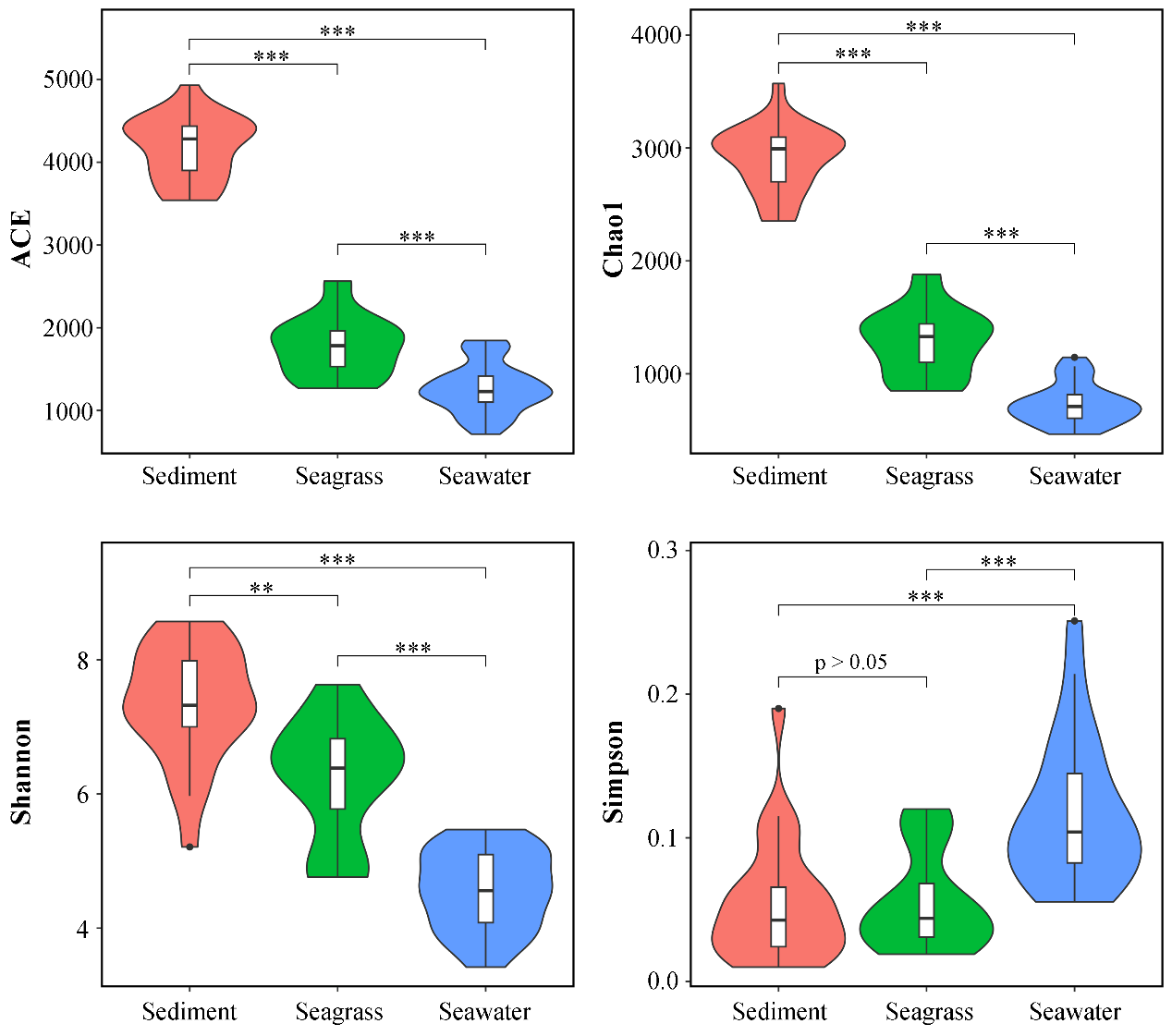


**Figure S1.** Violin plot revealed the difference of α-diversity index, including ACE, Chao1, Shannon, and Simpson, among the habitats of sediment, seagrass leaves and seawater. Index in each habitat contained the samples from all sampling sites. *, **, and *** denote significant effects at 0.01 < p ≤ 0.05, 0.001 < p ≤ 0.01, and p < 0.001, respectively.


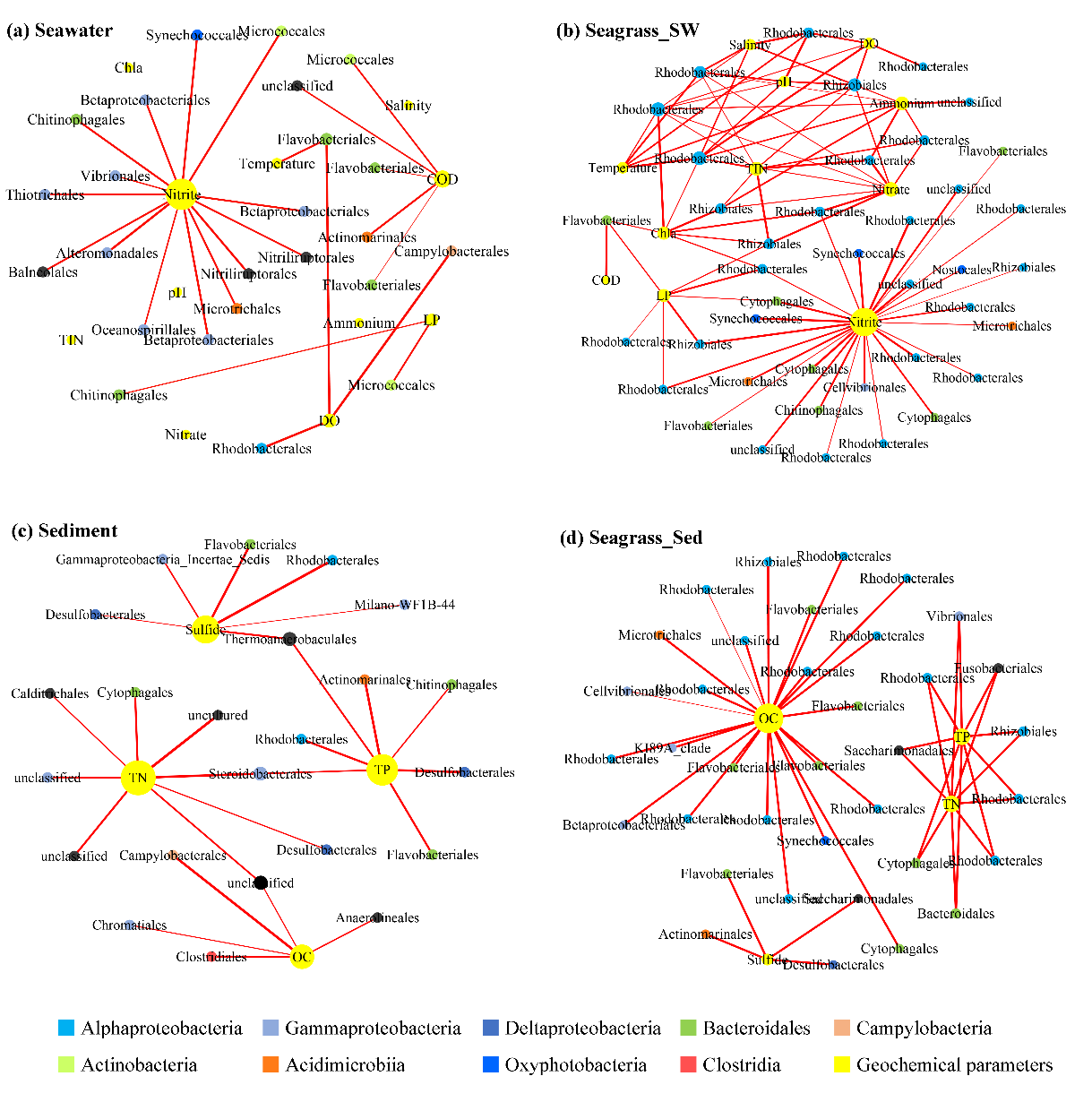


**Figure S2.** Network analysis of correlations between the 100 most abundant OTUs and physicochemical parameters within (a) seawater and (c) sediment, and correlations between the phylloshpere 100 most abundant OTUs and physicochemical parameters of (b) seawater and (d) sediment. The yellow nodes represent the geochemical parameters, the color of the OTUs were divided by the phylogenetic classification (at the class level) of OUTs. Size of the nodes were determined by the number of connections of each node with other nodes. Red and lines denote positive correlations between OTUs and geochemical parameters. The line width determined by the r value of Pearson test. Only correlations with absolute value of r > 0.6 and p < 0.05 were retained as valid co-occurrence events. Taxonomy for each OTU was given at order level. DO, dissolved oxygen; COD, chemical oxygen demand; TIN, total inorganic nitrogen; LP, labile phosphorus; OC, organic carbon; TP, total phosphorus; TN, total nitrogen.


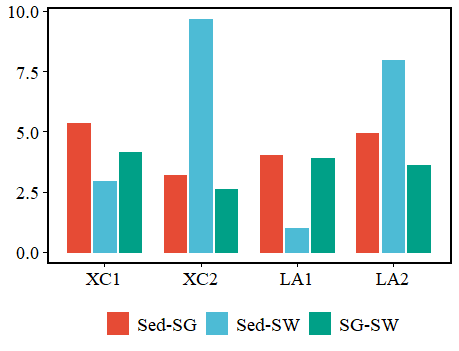


**Figure S3.** The total relative abundance of the connecters OTUs between the two habitats at each site.

Table S1. F values of two-way ANOVA tests showing the effects of lagoon and sampling site on α-diversity of microbial community.

| Type | Diversity index | Lagoon | Site | Lagoon × Site |
| --- | --- | --- | --- | --- |
| Sediment | ACE | 12.822** | 0.005 | 0.736 |
|  | Chao1 | 2.196 | 0.224 | 0.092 |
|  | Shannon | 11.057* | 0.002 | 2.201 |
|  | Simpson | 10.083* | 0.812 | 2.38 |
| Seagrass | ACE | 18.424** | 0.421 | 0.017 |
|  | Chao1 | 19.864** | 0.613 | 0.022 |
|  | Shannon | 10.736* | 0.203 | 0.107 |
|  | Simpson | 5.636* | 0.039 | 0.162 |
| Seawater | ACE | 3.551 | 5.139 | 0.475 |
|  | Chao1 | 3.273 | 5.453* | 0.944 |
|  | Shannon | 2.303 | 17.102** | 2.224 |
|  | Simpson | 4.917 | 17.601** | 5 |

* and ** denote significant effects at 0.01 < p ≤ 0.05 and 0.001 < p ≤ 0.01 respectively.

Table S2. Two-way permutational multivariate ANOVA and multivariate homogeneity test results, based on Bray–Curtis distances of microbial community composition between groups

| Habitats | Comparison | two-way PERMANOVA | | | Multivariate homogeneity Test | |
| --- | --- | --- | --- | --- | --- | --- |
|  |  | R^2^ | F | p | F | p |
| Sediment | Lagoon | 0.36543 | 9.2651 | 0.001 | 1.9506 | 0.61 |
|  | Site | 0.15075 | 3.822 | 0.022 | 0.3565 | 0.06 |
|  | Lagoon*Site | 0.16829 | 4.2669 | 0.009 | 5.5824 | ND |
| Seawater | Lagoon | 0.35464 | 13.9029 | 0.001 | 0.0988 | 0.63 |
|  | Site | 0.29098 | 11.4072 | 0.001 | 0.3565 | 0.064 |
|  | Lagoon*Site | 0.15031 | 5.8925 | 0.002 | 5.5824 | ND |
| Seagrass | Lagoon | 0.37397 | 6.3355 | 0.001 | 0.021 | 0.943 |
|  | Site | 0.07656 | 1.297 | 0.213 | 0.021 | 0.58 |
|  | Lagoon*Site | 0.07724 | 1.3086 | 0.221 | 0.3588 | ND |

Table S3. Network parameters in different sites

| Network attributes | XC1 | XC2 | LA1 | LA2 |
| --- | --- | --- | --- | --- |
| Nodes | 500 | 500 | 500 | 500 |
| Edges | 44312 | 37036 | 34478 | 32148 |
| Average degree | 88.624 | 74.072 | 68.956 | 64.296 |
| Diameter | 7 | 12 | 8 | 10 |
| Average path length | 2.33 | 4.026 | 2.674 | 2.936 |
| Density | 0.178 | 0.148 | 0.138 | 0.129 |
| Average clustering coefficient | 0.751 | 0.737 | 0.753 | 0.743 |
| Negative | 2.30% | 1.00% | 0.40% | 0.30% |

Table S4. The nodes, edges and average degree of the clusters in each network

| Site | Cluster | Nodes | Edges | Average degree |
| --- | --- | --- | --- | --- |
| XC1 | #1 combined 2 | 275 | 36430 | 133 |
|  | #3 | 46 | 848 | 18 |
|  | #4 | 108 | 5446 | 50 |
|  | #5 | 61 | 1586 | 26 |
| XC2 | #1 combined 2 | 265 | 29965 | 113 |
|  | #3 | 69 | 1592 | 23 |
|  | #4 | 91 | 4435 | 49 |
|  | #5 | 50 | 856 | 17 |
| LA1 | #1 combined 2 | 211 | 21368 | 101 |
|  | #3 | 130 | 6302 | 49 |
|  | #4 | 88 | 4722 | 54 |
|  | #5 | 61 | 1720 | 28 |
| LA2 | #1 combined 2 | 205 | 18681 | 91 |
|  | #3 | 149 | 9154 | 61 |
|  | #4 | 59 | 2703 | 46 |
|  | #5 | 66 | 1572 | 24 |

Table S5. The number of links between two clusters or two habitats and percentage of the links to total edges within the two clusters

| Cluster link | Links between clusters | | | | % of links to total edges | | | |
| --- | --- | --- | --- | --- | --- | --- | --- | --- |
|  | XC1 | XC2 | LA1 | LA2 | XC1 | XC2 | LA1 | LA2 |
| 3−4 | 74 | 19 | 364 | 370 | 1.2 | 0.3 | 3.3 | 3.2 |
| 1−2 | 5358 | 3964 | 2065 | 2458 | 17.2 | 15.2 | 10.7 | 15.2 |
| (3+4)−(1+2) | 0 | 4 | 0 | 0 | 0 | 0 | 0 | 0 |
| (3+4)−5 | 0 | 0 | 0 | 1 | 0 | 0 | 0 | 0 |
| (1+2)−5 | 8 | 2 | 2 | 0 | 0 | 0 | 0 | 0 |

1. *Corresponding author:

   Guangcheng Chen

   gc.chen@tio.org.cn

   Yao Zhang

   yaozhang@xmu.edu.cn [↑](#footnote-ref-1)
